# Characterizing Spatial Patterns of Airborne Coarse Particulate (PM_10–2.5_) Mass and Chemical Components in Three Cities: The Multi-Ethnic Study of Atherosclerosis

**DOI:** 10.1289/ehp.1307287

**Published:** 2014-03-18

**Authors:** Kai Zhang, Timothy V. Larson, Amanda Gassett, Adam A. Szpiro, Martha Daviglus, Gregory L. Burke, Joel D. Kaufman, Sara D. Adar

**Affiliations:** 1 Division of Epidemiology, Human Genetics and Environmental Sciences, University of Texas School of Public Health, Houston, Texas, USA; 2 Department of Civil and Environmental Engineering,; 3 Department of Environmental and Occupational Health Sciences, and; 4 Department of Biostatistics, University of Washington, Seattle, Washington, USA; 5 Department of Medicine, University of Illinois at Chicago, Chicago, Illinois, USA; 6 Division of Public Health Sciences, Wake Forest University School of Medicine, Winston-Salem, North Carolina, USA; 7 Department of Epidemiology, University of Washington, Seattle, Washington, USA; 8 Department of Epidemiology, University of Michigan, Ann Arbor, Michigan, USA

## Abstract

Background: The long-term health effects of coarse particular matter (PM_10–2.5_) are challenging to assess because of a limited understanding of the spatial variation in PM_10–2.5_ mass and its chemical components.

Objectives: We conducted a spatially intensive field study and developed spatial prediction models for PM_10–2.5_ mass and four selected species (copper, zinc, phosphorus, and silicon) in three American cities.

Methods: PM_10–2.5_ snapshot campaigns were conducted in Chicago, Illinois; St. Paul, Minnesota; and Winston-Salem, North Carolina, in 2009 for the Multi-Ethnic Study of Atherosclerosis and Coarse Airborne Particulate Matter (MESA Coarse). In each city, samples were collected simultaneously outside the homes of approximately 40 participants over 2 weeks in the winter and/or summer. City-specific and combined prediction models were developed using land use regression (LUR) and universal kriging (UK). Model performance was evaluated by cross-validation (CV).

Results: PM_10–2.5_ mass and species varied within and between cities in a manner that was predictable by geographic covariates. City-specific LUR models generally performed well for total mass (CV *R*^2^, 0.41–0.68), copper (CV *R*^2^, 0.51–0.86), phosphorus (CV *R*^2^, 0.50–0.76), silicon (CV *R*^2^, 0.48–0.93), and zinc (CV *R*^2^, 0.36–0.73). Models pooled across all cities inconsistently captured within-city variability. Little difference was observed between the performance of LUR and UK models in predicting concentrations.

Conclusions: Characterization of fine-scale spatial variability of these often heterogeneous pollutants using geographic covariates should reduce exposure misclassification and increase the power of epidemiological studies investigating the long-term health impacts of PM_10–2.5_.

Citation: Zhang K, Larson TV, Gassett A, Szpiro AA, Daviglus M, Burke GL, Kaufman JD, Adar SD. 2014. Characterizing spatial patterns of airborne coarse particulate (PM_10–2.5_) mass and chemical components in three cities: the Multi-Ethnic Study of Atherosclerosis. Environ Health Perspect 122:823–830; http://dx.doi.org/10.1289/ehp.1307287

## Introduction

Although considerable evidence has linked adverse health with long-term exposures to fine particulate matter (PM_2.5_; ≤ 2.5 μm in aerodynamic diameter) (Brook et al. 2010), there has been little epidemiological research examining long-term exposures to coarse particulate matter (PM_10–2.5_; 2.5–10 μm in aerodynamic diameter). Toxicological studies have shown that PM_10–2.5_ can induce reactive oxygen species and initiate inflammatory responses *in vivo* and *in vitro* (Becker et al. 2005; Monn and Becker 1999; Pozzi et al. 2003; Schins et al. 2004; Shi et al. 2003), suggesting a plausible biological mechanism for long-term health effects. The studies that have investigated such relationships with PM_10–2.5_, however, have generally found weak and nonstatistically significant or null associations [U.S. Environmental Protection Agency (EPA) 2006]. One possible explanation for differences between the toxicological and epidemiological evidence is that previous epidemiological studies have had a limited ability to characterize spatial variations in PM_10–2.5._ This can be important because PM_10–2.5_ has relatively short residence times in atmosphere due to high gravitational settling (U.S. EPA 2009), and spatial heterogeneity has been shown to be large (Burton et al. 1996; Chen et al. 2007; Eeftens et al. 2012a; Houthuijs et al. 2001; Wilson and Suh 1997). In addition, there has been limited characterization of spatial differences in PM_10–2.5_ chemical composition, which may help to differentiate key sources of PM_10–2.5_ mass (e.g., mineral and roadway dust, sea spray, pollen, and mechanical grinding including vehicular brake and tire wear) (U.S. EPA 2009). An improved understanding of the spatial variation of PM_10–2.5_ mass and chemical components is therefore expected to be critical in quantifying the long-term effects of PM_10–2.5_ exposures.

Because some pollutants, such as those from traffic, vary over small spatial scales (i.e., 10–100 m), there is an increasing emphasis on estimating individual-level exposures (Health Effects Institute 2010). Regression models with geographic information system (GIS)–derived covariates such as land use, nearby emission sources, and distance to roadways, termed “land use” regression (LUR) models, are a common approach. Universal kriging (UK) is an extension of this methodology that further incorporates spatial correlations. Although spatial prediction models are commonly employed for PM_2.5_ and oxides of nitrogen (Health Effects Institute 2010), very few investigations have generated covariate-based spatial prediction models for PM_10–2.5_, and none, to our knowledge, have created covariate-based, spatial interpolation models for PM_10–2.5_ species.

As part of the Multi-Ethnic Study of Atherosclerosis and Coarse Airborne Particulate Matter (MESA Coarse), we characterized fine-scale spatial differences in PM_10–2.5_ mass and chemical components within three American cities using data from intensive monitoring campaigns. MESA Coarse builds on the MESA cohort of 6,814 adults from six metropolitan areas (Bild et al. 2002) and the MESA Air Pollution project (MESA Air), which investigates the impacts of PM_2.5_ on the progression of atherosclerosis (Cohen et al. 2009; Kaufman et al., 2012). Here we present the MESA Coarse field study design and development of spatial prediction models for PM_10–2.5_ mass, copper, zinc, phosphorus, and silicon. These four chemical components were selected because they were shown to be good indicators of brake wear, tire wear, agriculture, and mineral dust, respectively, across all three cities, using positive matrix factorization (Sturtz et al. 2014).

## Methods

*Sampling design*. PM_10–2.5_ concentrations were measured simultaneously over 2-week periods in two seasons outside the homes of approximately 40 MESA participants residing in Chicago, Illinois (8–22 April 2009; 20 August–3 September 2009), St. Paul and Minneapolis, Minnesota (17–31 January 2009; 27 May–10 June 2009), and Winston-Salem, North Carolina (25 February–11 March 2009; 6–20 July 2009). Homes were selected in a targeted approach that aimed to maximize geographic coverage as well as variability of features believed to be predictive of coarse particles and selected source-specific components. Specifically, we targeted vegetation, distance to major roads, as well as rural, commercial, and industrial land use. Although most homes were sampled during one season only, we collected samples during both seasons at approximately one-third of homes to assess the stability of concentrations over time. Homes with more unique geographic features were oversampled during the second round to ensure sufficient variability for modeling. Other repeats were selected at random. Institutional review boards at each site approved the study, and all participants provided written informed consent.

*PM_10–2.5_ sampling*. We collected 2-week integrated samples using Harvard personal environmental monitors (HPEMs; Thermo Environmental Instruments, Franklin, MA) with Medo VP0125 pumps (Medo, Hanover Park, IL) calibrated to a flow rate of 1.8 L/min, which has been evaluated in ambient field tests against the Harvard impactor operating at 10 L/min (Lee et al. 2006). To prevent overloading and minimize the number of pumps required, air flow was cycled between paired HPEMs with cut points for PM_10_ and PM_2.5_ every 5 min over the 2-week sampling periods. Programmable timers allowed for the simultaneous collection of samples across all locations in a city.

All Teflon filters were preconditioned for ≥ 24 hr at 22.3 ± 1.9°C and 34.7 ± 2.5% relative humidity, before weighing by microbalance (model UMT2; Mettler-Toledo Inc., Highstown, NJ) (Allen et al. 2001). Samples were analyzed for elements by X-ray fluorescence spectroscopy by Cooper Environmental Services (Portland, OR). Concentrations were estimated by subtracting PM_2.5_ from PM_10_ based on research by Chen et al. (2011).

Many quality control procedures were performed including voiding samples with insufficient durations (< 9 days), out-of-range air flows (± 20%), damaged filters, extreme concentrations over the 2-week sampling period (> 5 SDs from the mean), and high sulfur levels in the PM_10–2.5_ fraction (> 0.2 μg/m^3^) because sulfur should be limited primarily to PM_2.5_. Overall, the precision of duplicate PM_10_, PM_2.5_, and PM_10–2.5_ samples was 2%, 10%, and 18%, respectively. Concentrations were also compared with measurements reported by the U.S. EPA’s Air Quality System (AQS) for corresponding time periods during the same year (U.S. EPA 2010).

*Geographic covariates*. Table 1 illustrates the covariates derived in ArcGIS 9.3 (ESRI, Redlands, CA) that were considered for our spatial prediction models. They include five major categories: *a*) land use such as commercial, industrial, and residential; *b*) local transportation including roadways, railways, truck routes, airports, and a traffic dispersion model output; *c*) population density; *d*) ground cover including impervious surface and vegetation; *e*) PM_10–2.5_ emission sources; and *f*) positional information (MESA Air 2011). Briefly, land use data included U.S. Geological Survey satellite-derived raster images from 2000 (Price et al. 2006) and aerial photography from the 1970s and 1980s.Transportation variables were derived from data from TeleAtlas (Lebanon, NH), National Transportation Atlas Database 2009 (Bureau of Transportation Statistics 2009), and the CALINE3 line source dispersion model (Wilton et al. 2010). PM_10–2.5_ emissions were derived from the National Emission Inventory Database (U.S. EPA 2008), population density was obtained from the 2000 U.S. Census (U.S. Census Bureau 2000), imperviousness was downloaded from the National Land Cover Database 2006 (U.S. Geological Survey 2011), and vegetation was estimated by the Normalized Difference Vegetation Index (NDVI) (Carroll et al. 2008).

**Table 1:** Variables considered for spatial prediction models for PM_10–2.5_ mass and chemical component concentrations.

Variable	Unit	Buffer radii
Land use: satellite based
Open water; developed open space; developed low intensity; developed medium intensity; developed high intensity; bare rock/sand/barren/mine; trees; shrub land; grasslands/herbaceous vegetation; pasture/hay; cultivated crops; woody wetlands.	Percent	50, 100, 150, 300, 400, 500, 750, 1,000, 1,500, 3,000, 5,000 m
Land use: aerial photography based
Residential; commercial and services; industrial, transportation, communications, and utilities; other urban or built-up land; mixed urban or built-up land; strip mines, quarries, and gravel pits; industrial and commercial complexes; transitional areas.	Percent	50, 100, 150 m
Local transportation
Distance to the nearest A1/A2/A3 road	Meters	NA
Length of A1/A2/A3 roads in buffers	Meters	50, 100, 150, 300, 400, 500, 750, 1,000, 1,500, 3,000, 5,000 m
Distance to the nearest truck route/railroad/rail yard/airport/large port	Meters
Length of truck routes in buffers	Meters	100, 150, 300, 400,500, 750, 1,000, 1,500, 3,000, 5,000, 10,000, 15,000 m
CALINE long-term average^*a*^	NA	1.5, 3, 4.5, 6, 7.5, 9 km
Population density	Person/km^2^	3, 5, 10, 15 km
Ground cover
Imperviousness	Percent	50, 100, 150, 300, 400, 500, 750, 1,000, 3,000, 5,000 m
NDVI in the 25th and 75th percentiles	NA	250, 500, 1,000, 5,000 m
Sum PM_10–2.5_ emissions	Tons/year	< 3, 3–15, 3–30 km
Positional information
Latitude and longitude (X,Y)	Meters	NA
Distance to main and local city hall	Meters	NA
Abbreviations: NA, not applicable; NDVI, Normalized Difference Vegetation Index. ^***a***^CALINE3 long-term average concentrations of a traffic-generated inert gaseous pollutant were generated from a line source dispersion model (Wilton et al. 2010).		

*Modeling approach*. We used LUR and UK to estimate spatial patterns of PM_10–2.5_ mass, copper, zinc, phosphorus, and silicon using approaches previously described by Mercer et al. (2011). Our primary models were constructed separately for each city, with season included as a predictor and an effect modifier of other predictors as needed to account for seasonal variation. Pooled models across all cities were also explored in secondary analyses. All stages of our model selection procedure were fit using the glmnet package (Friedman et al. 2010) and R 2.7.2 software (R Development Core Team; http://R-project.org). The resulting prediction models are intended to reflect long-term exposures because preliminary data analyses using AQS monitors in our study regions previously suggested that the average of 2-week samples from two seasons was highly correlated with annual average concentrations.

Given the large number of potential predictors, we followed a process of variable screening that began with removal of predictors with insufficient variability (i.e., excluded if 85th percentile equaled the 15th percentile). Then, for variables with varying buffer radii, we selected the best short-range (50–500 m) and long-range (500 m to 5 km) buffer based on the highest univariate Pearson correlation coefficient with the exposure being modeled. Moreover, when multiple predictors were highly correlated with one another (ρ > 0.85), we selected the predictor that was most strongly correlated with the exposure, although we preferentially excluded latitude and longitude and selected raster-based land use data (collected in 2000) over older aerial photography-based data (collected in the 1970s and 1980s). Next, we applied the least absolute shrinkage and selection operator (LASSO) by changing a tuning parameter to reduce the number of variables down to ≤ 15 (Friedman et al. 2010). Finally, we conducted an exhaustive search to examine all possible combinations of these covariates, restricting to models with ≤ 6 parameters. When interaction terms were selected by either of these modeling steps without the main effects, we also forced the main effects into the model. No other interactions between predictors other than season or city were considered.

The final combinations of variables were selected that resulted in the lowest root-mean-square error (RMSE) and the highest *R*^2^ under 10-fold cross-validation (CV). In this method, the data set was randomly divided into 10 equal sub–data sets, where model fitting occurred for each selection of nine-tenths of the data while validating on the final tenth. Differences between the true and estimated values of PM_10–2.5_ concentrations for each validation set were then used to calculated RMSE and *R*^2^. This method is intended to avoid overfitting of the models to the observed data.

Sensitivity analyses were conducted to assess the impacts of *a*) excluding outliers (> 3 SDs from city-specific means); *b*) different data sources of land use variables; *c*) selecting buffers in a repeated step-wise manner, as recommended by Su et al. (2009); and *d*) natural log-transforming concentrations.

*Visualization*. Maps were generated by kriging provided by the spatial analysis package in ArcGIS 9.3 at a lattice grid over our three cities, with spacing of 0.25 km in urban areas and 1–2 km in rural areas.

## Results

**Figure 1:**
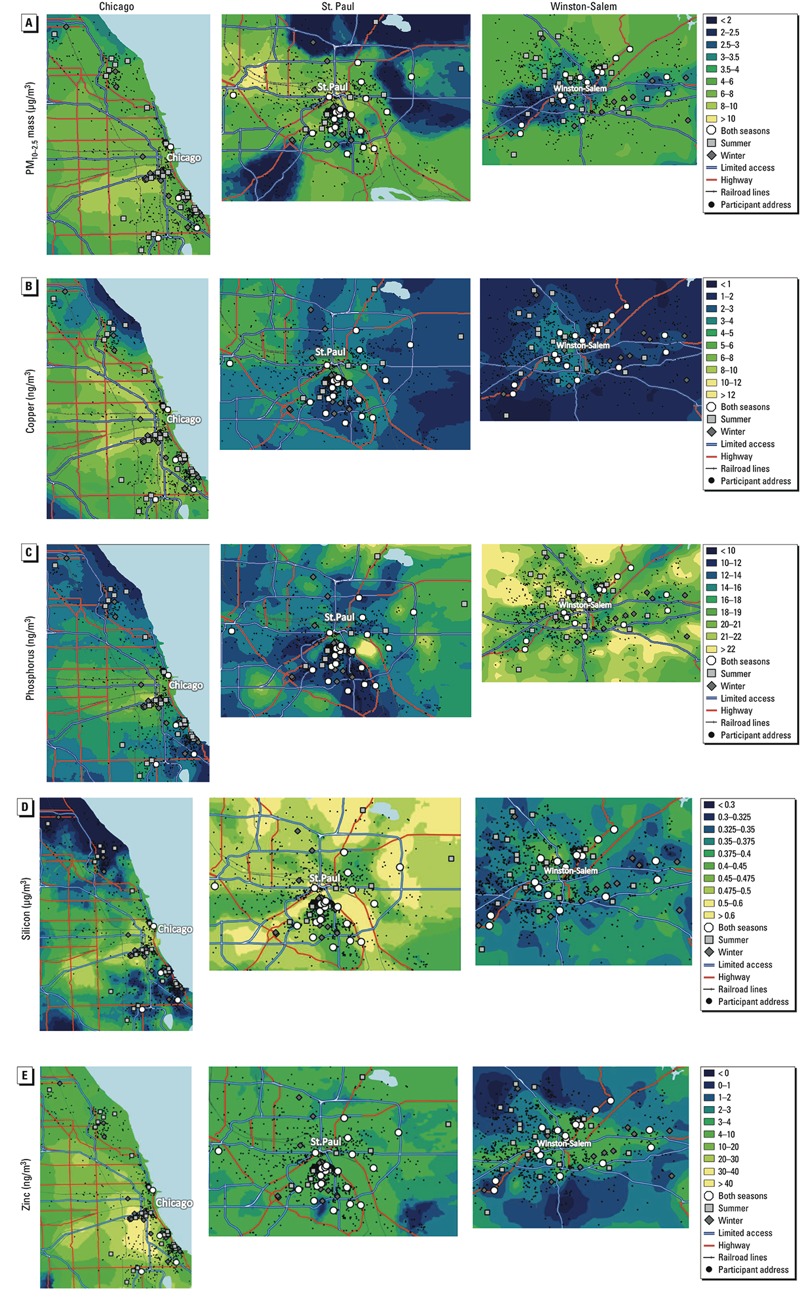
Predicted concentrations for PM_10–2.5_ mass (*A*), copper (*B*), phosphorus (*C*), silicon (*D*), and zinc (*E*) in three U.S. cities. Bins were chosen to highlight within-city contrasts and are not even. Participants’ locations have been jittered.

*Measured PM_10–2.5_ mass concentrations*. Between 17 January and 3 September 2009, we collected 235 collocated PM_2.5_ and PM_10_ samples. After our quality control procedures were applied, we had 207 (88%) and 195 (83%) valid PM_2.5_ and PM_10_ mass measurements, respectively, resulting in 191 (81%) valid sample pairs from 156 unique locations (61, 43, and 52 in Chicago, St. Paul, and Winston-Salem, respectively; Figure 1). A subset of 35 locations had samples collected during two seasons: 4, 18, and 13 in Chicago, St. Paul, and Winston-Salem, respectively.

Table 2 summarizes PM_10–2.5_ mass and species concentrations by city and season (see Supplemental Material, Table S1, for detailed descriptive statistics). Mean (± SD) PM_10–2.5_ concentrations across seasons were 5.7 ± 2.0, 5.3 ± 3.3, and 3.6 ± 1.4 μg/m^3^ in Chicago, St. Paul, and Winston-Salem, respectively. A strong seasonal difference was seen in St. Paul (3.3 ± 2.2 and 6.7 ± 3.3 μg/m^3^ in winter and summer, respectively) but not in the other two cities (Chicago: 5.5 ± 2.0 and 5.9 ± 2.1 μg/m^3^ in winter/early spring and summer, respectively; Winston-Salem: 3.5 ± 1.2 and 3.8 ± 1.6 μg/m^3^ in winter and summer, respectively).

**Table 2:** Summary of statistics (mean ± SD) for PM_10–2.5_ mass (μg/m^3^) and chemical component (ng/m^3^)*^a^* concentrations in each sampling city by season.

City and season^*b*^	*n*	Total mass	Copper	Phosphorus	Silicon	Zinc
Chicago, IL
Winter	33^*b*^	5.54 ± 1.98	7.83 ± 3.32	13.64 ± 6.00	0.43 ± 0.11	23.74 ± 18.36
Summer	31	5.94 ± 2.09	7.10 ± 4.37	17.87 ± 3.87	0.31 ± 0.16	25.87 ± 22.85
Pooled	64^*c*^	5.73 ± 2.03	7.47 ± 3.86	15.72 ± 5.46	0.37 ± 0.15	24.79 ± 20.55
St. Paul, MN
Winter	25	3.34 ± 2.22	4.01 ± 1.23	8.20 ± 4.68	0.27 ± 0.04	5.23 ± 3.42
Summer	34	6.66 ± 3.33	2.77 ± 1.69	18.67 ± 5.44	0.72 ± 0.19	5.55 ± 7.03
Pooled	59	5.25 ± 3.33	3.29 ± 1.63	14.23 ± 7.29	0.53 ± 0.27	5.42 ± 5.74
Winston-Salem, NC
Winter	35	3.46 ± 1.21	2.57 ± 1.23	12.83 ± 3.70	0.41 ± 0.09	3.31 ± 2.67
Summer	28	3.83 ± 1.64	2.57 ± 1.46	25.90 ± 5.71	0.35 ± 0.11	2.76 ± 1.95
Pooled	63	3.63 ± 1.42	2.57 ± 1.33	18.64 ± 8.04	0.38 ± 0.10	3.07 ± 2.37
^***a***^Silicon values in μg/m^3^. ^***b***^All sampling was conducted in 2009. Chicago: winter (April 8–22), summer (August 20–September 3); St. Paul: winter (January 17–31), summer (May 27–June 10); Winston-Salem: winter (February 25–March 11), summer (July 6–20). ^***b***^Thirty-two for the four species. ^***c***^Sixty-three for the four species.						

Silicon had the largest concentrations of the species investigated, with levels 12 to 260 times larger than the three other species (Table 2). Especially high concentrations of silicon were observed in St. Paul during the summer (720 ± 188 ng/m^3^), whereas the other two cities had lower levels that peaked in the winter. Phosphorus concentrations in all three cities were highest in the summer, with an approximate doubling of concentrations compared with the winter in St. Paul and Winston-Salem. In contrast to the other pollutants that had similar concentration ranges across cities, copper and zinc concentrations differed by location, with the highest levels of both observed in Chicago, and the lowest levels in Winston-Salem. Zinc was the most variable species in each of the three cities during both the winter (coefficient of variation defined as SD over mean: 0.75–0.81) and summer (coefficient of variation: 0.69–1.27).

*Spatial modeling results*. Data reduction procedures reduced the overall number of potential predictors from 802 to 64–94, and the LASSO procedure further reduced the number of candidate predictors to approximately 15 for each city and species. Final models included seven or eight main effect predictors and up to four interactions (with season in the city-specific models, and with season or city in the all-city models) (see Supplemental Material, Tables S2–S6, for lists of the predictors included in each final model according to city and exposure.).

**Figure 2:**
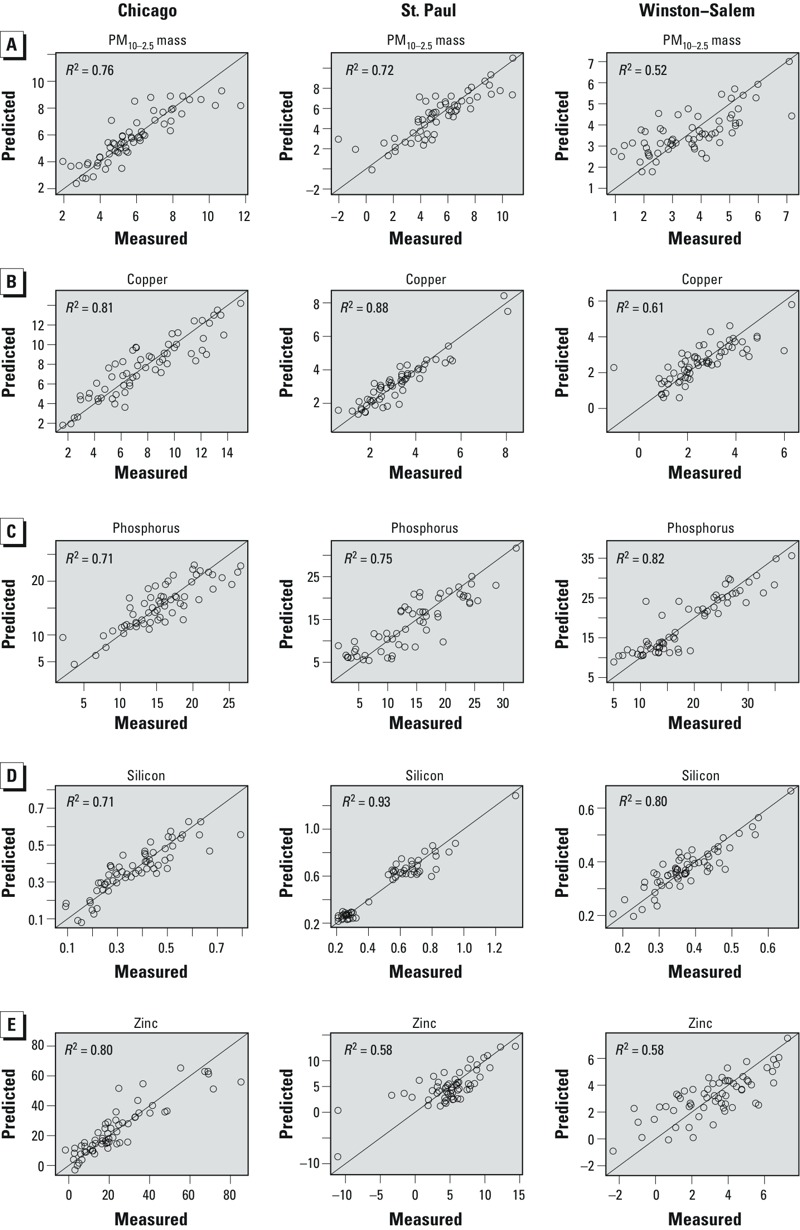
Scatter plots showing observations and the predictions from the “best” LUR ­models by city and species: PM_10–2.5_ (*A*), copper (*B*), phosphorus (*C*), silicon (*D*), and zinc (*E*). *R*^2^ = square of correlations between measurements and predictions.

Overall, our models fit the data well (Figure 2), explaining between 36% and 93% of the variability in PM_10–2.5_ mass and species concentrations under cross-validation (Table 3). Our models performed generally better in Chicago and St. Paul than in Winston-Salem, and had the most consistent predictive ability for silicon when including data for all cities. The other models pooled across all cities demonstrated inconsistent performance at capturing within-city variability, with the best within-city CV *R*^2^ ranging from 0.54 to 0.66 compared with 0.0 to 0.34 for cities with the worst predictions. UK models for individual cities as well as pooled across all cities generally had similar or lower model performance than their corresponding LUR models.

**Table 3:** Model performance (cross-validated *R*^2^ and RMSE) for PM_10–2.5_ mass (μg/m^3^) and species concentrations (ng/m^3^)*^a^* using land use regression (LUR) and universal kriging (UK) models.

Model	CV measure	Total mass	Copper	Phosphorus	Silicon	Zinc
Land use regression^*b*^
Chicago, IL	*R*^2^	0.68	0.65	0.50	0.68	0.73
Chicago, IL	RMSE	1.16	2.29	3.88	0.08	10.63
St Paul, MN	*R*^2^	0.51	0.86	0.68	0.93	0.40
St Paul, MN	RMSE	2.33	0.61	4.14	0.07	4.44
Winston Salem, NC	*R*^2^	0.41	0.51	0.76	0.48	0.36
Winston Salem, NC	RMSE	1.09	0.93	3.95	0.07	1.89
All cities^*b*^	*R*^2^	0.52, 0.54, 0.10	0.65, 0.49, 0.09	0.34, 0.59, 0.66	0.24, 0.64, 0	0.61, 0, 0
All cities^*b*^	RMSE	1.39, 2.24, 1.33	2.26, 1.06, 1.25	4.39, 4.63, 4.66	0.13, 0.16, 0.12	12.72, 6.10, 3.58
Universal kriging
Chicago, IL	*R*^2^	0.68	0.64	0.50	0.68	0.73
Chicago, IL	RMSE	1.14	2.32	3.88	0.08	10.60
St Paul, MN	*R*^2^	0.51	0.86	0.68	0.91	0.38
St Paul, MN	RMSE	2.32	0.61	4.14	0.08	4.52
Winston Salem, NC	*R*^2^	0.41	0.51	0.76	0.47	0.36
Winston Salem, NC	RMSE	1.09	0.93	3.95	0.07	1.89
All cities	*R*^2^	0.51, 0.52, 0.11	0.20, 0.20, 0	0.15, 0.38, 0.65	0.22, 0.64, 0	0, 0, 0
All cities	RMSE	1.42, 2.29, 1.33	3.42, 1.34, 2.10	5.00, 5.67, 4.78	0.13, 0.16, 0.13	22.01, 7.66, 10.58
^***a***^Silicon values in μg/m^3^. ^***b***^The predictors included in each model are listed in Supplemental Material, Tables S2–S6. ^***b***^For pooled models across all cities, we present the *R*^2^ for the explanatory power of each city as calculated by the formula (1 – sum of squared differences between observations and predictions/sum of squared differences between observations and city-specific means). *R*^2^ with values < 0 are reported as 0. RMSE was calculated as the square root of the average of squared differences between observations and predictions. The respective *R*^2^ and RMSE values for each city appear in the following order: Chicago, St. Paul, Winston-Salem.						

Figure 1 shows the spatial patterns and distributions of predicted concentrations by city. PM_10–2.5_ mass, silicon, and phosphorus had somewhat similar prediction ranges across all cities, whereas copper and zinc showed much higher predictions in Chicago and St. Paul than in Winston-Salem. The highest PM_10–2.5_ mass predictions were for the urban centers of Chicago and St. Paul, but in Winston-Salem higher levels were predicted outside of the urban core. Across all three cities, copper and zinc exhibited higher concentrations in urban areas, with elevations of copper focused in areas of high-intensity land use and along major roadways. Silicon was highest in St. Paul and most variable in Chicago, where high concentrations were focused along an industrial corridor to the west of the city and lowest concentrations were found in the outlying areas. Phosphorus was similarly patterned in Chicago but less concentrated downtown. Predictions were highly variable for phosphorus in St. Paul but consistently high in Winston-Salem, with higher predictions in the outlying areas.

The estimated coefficients of the LUR models by city and pooled across cities are available in the Supplemental Material, Tables S2–S6. In general, PM_10–2.5_ mass, copper, and zinc concentrations were associated with traffic-related features across the three cities (e.g., land use, distance to roads, sum of road lengths, and sum of truck route lengths). Models for phosphorus and silicon concentrations consistently included vegetation features as predictors in St. Paul and Winston-Salem, but also included water features in Chicago. Vegetation appeared predictive across all mass and species models in Winston-Salem. Medium or high intensity of urban land use was predictive among all models for total mass, and sum of road lengths was consistently included in all pooled models, as were season and city.

Sensitivity analyses indicated that two extreme measurements from St. Paul were influential, with weaker predictive performance without the outliers (CV *R*^2^, 0.51 compared with 0.65). Slightly improved model performance was found using land use from both the raster data and aerial photography, compared with either alone (see Supplemental Material, Table S7). Models based on the natural log of PM_10–2.5_ levels and the iterative covariate selection approach proposed by Su et al. (2009) showed generally poorer prediction performance as denoted by lower CV *R*^2^ and higher RMSE than those presented here (data not shown).

## Discussion

In this study we conducted spatially intensive PM_10–2.5_ sampling and developed two types of spatial prediction models (LUR and UK) for PM_10–2.5_ mass and chemical components (copper, phosphorus, silicon, and zinc) for use in the MESA Coarse epidemiology study. To our knowledge, this is one of the first studies to develop fine-scale spatial prediction models for PM_10–2.5_ mass and chemical components, which can serve as tracers for different sources of pollution. We demonstrated that geographic covariates can explain within-city variations in concentrations (mean CV *R*^2^, 0.61), although the predictive power varies across cities and species (range of CV *R*^2^, 0.36–0.93). By capturing fine-scale spatial variability of these often heterogeneous pollutants, this work is expected to substantially reduce measurement error and improve our ability to investigate the long-term health impacts of PM_10–2.5_ over traditional approaches that rely on a limited number of central monitoring stations.

Across all models, there was some similarity in the spatial features predictive of concentrations, including high- and medium-intensity land use and indicators of traffic and vegetation, yet there was limited commonality across cities in the key predictors. For example, variables related to traffic, high/medium development, and residential areas were consistently selected in final models in Chicago, but indicators of vegetation were more consistently included in final models in Winston-Salem. This may reflect the fact that Chicago is a more industrialized city, with higher concentrations of copper and zinc, than Winston-Salem, which is a smaller city. Similarly, water features appeared to be a stronger predictor of PM_10–2.5_ particles in St. Paul than the other cities likely because this is a prominent feature of this region.

Season modified the influence of certain predictors, as would be expected given the presence of snow cover during winter periods in St. Paul and Chicago and changes in vegetation across all areas. Although other characteristics of seasonality may influence PM_10–2.5_ concentrations, we did not have fine-scale spatial meteorology data to explicitly explore these associations. An analysis of wind speed and direction, however, failed to show a strong prevailing wind direction during our sampling periods, suggesting that wind is likely not a strong predictor or modifier of concentrations. In addition, we did not identify important differences between our sampling periods and other typical weeks, suggesting that our results should be representative of other time periods.

Given the observed area-specific differences, we were unable to identify robust predictions of within-city variability based on the same models for all cities (see Supplemental Material, Tables S2–S6). Although some of our pooled models showed good performance in more than one city, no model worked well in all locations. Thus, though it is possible that these models may be generalized to other cities with similar characteristics, further validation is warranted before broad application.

Predictive performance of our models was consistent with the one study from Europe (Eeftens et al. 2012a) and generally better than the few PM_10–2.5_ models in the United States. Using 20–40 monitoring stations in each of 20 European areas, similar CV *R*^2^ values were reported as in our study (Eeftens et al. CV *R*^2^, 0.03–0.73; our MESA Coarse study CV *R*^2^, 0.41–0.68.) (Eeftens et al. 2012b). In a recent study from Ohio, Mukerjee et al. (2012) reported an *R*^2^ of 0.78 for their LUR model. Because this was not derived from cross-validation in which some data are withheld from the model-building step for validation, however, the predictive power would be inflated due to overfitting. Also in the United States, Yanosky et al. (2009) reported models with CV *R*^2^ values for PM_10–2.5_ of 0.39 and 0.33 after and before 1999 for cities across the northeastern and midwestern United States, respectively. Their lower predictive performance can be likely be explained by their use of regulatory monitors only, which are more sparse and reflect less variation in geographical predictors than the intensive campaigns of this, the European, and Ohio studies. Predicting PM_10–2.5_ indirectly by modeling PM_2.5_ and PM_10_ separately may also have influenced their models. Independent of the source of data, we generally found similar predictors to past work including proximity to roadways, land use, and vegetation. As in our study, Eeftens et al. (2012b) also indicated that key covariates differed by city.

UK performed similarly to LUR for all cities, suggesting limited spatial correlation of PM_10–2.5_ after control for geographic covariates. An alternative explanation is that our relatively small sample size (*n* ~ 30–40 locations in each city) may have limited the ability of our models to characterize fine-scale spatial structure of PM_10–2.5_. Nevertheless, our models should have been able to capture at least some of any small-scale correlation structure, given that distances between some of the sampled residences were small (minimum distances of 8, 64, and 174 m in Chicago, St. Paul, and Winston-Salem, respectively).

Measured PM_10–2.5_ levels as well as spatial variability were generally lower than expected, possibly because our study samples were collected exclusively at residual locations where there may be fewer sources of PM_10–2.5_. The average levels of PM_10–2.5_ measured at study locations in both Chicago and St. Paul (5–6 μg/m^3^) were comparable with those reported in residential neighborhoods in Detroit, Michigan (6–7 μg/m^3^) (Thornburg et al. 2009), but lower than reported for a variety of sites in Los Angeles, California (5–14 μg/m^3^) (Pakbin et al. 2010), Philadelphia, Pennsylvania (5–9 μg/m^3^) (Burton et al. 1996), Denver, Colorado (9–16 μg/m^3^) (Clements et al. 2012), Research Triangle Park, North Carolina (1–13 μg/m^3^) (Chen et al. 2007), and Central and Eastern European countries (12–40 and 6–24 μg/m^3^) (Eeftens et al. 2012a; Houthuijs et al. 2001). Although our means were lower, study locations with the highest measured concentrations (12 μg/m^3^ in Chicago; 17 μg/m^3^ in St. Paul) were in line with concentrations reported by these other studies.

Spatial variability also was lower than expected, which may also be attributable to sampling from residential locations only. Average PM_10–2.5_ concentrations were, however, generally comparable with those estimated by concurrent PM_10_ and PM_2.5_ AQS samples during the same 2-week sampling periods. For example, the 2-week averages of four available Chicago AQS sites were 4.5 ± 5.3 and 6.1 ± 2.1 μg/m^3^ when we reported 5.5 ± 2.0 μg/m^3^ and 5.9 ± 2.1 μg/m^3^ in matched time periods. In Winston-Salem, one available AQS site had one 2-week average of 3.7 ± 1.5 μg/m^3^ when we observed 3.8 ± 1.6 μg/m^3^, and 2.4 ± 0.2 μg/m^3^ when we observed 3.5 ± 1.2 μg/m^3^. In St. Paul, two available AQS sites had 2-week mean levels of 6.6 ± 4.7 and 8.6 ± 4.6 μg/m^3^ when our reported concentrations were 3.3 ± 2.2 and 6.7 ± 3.3 μg/m^3^, respectively.

This study has a few important strengths. First, we collected spatially intensive samples of PM_10–2.5_ using the same sampling protocol in three U.S. cities. Using a snapshot style campaign, we were able to predict PM_10–2.5_ mass at unmeasured locations based on geographic characteristics of that precise location. This represents a substantial improvement for predicting long-term concentrations at unmeasured locations compared with assigning the concentration of the nearest monitor or simple interpolation methods that do not consider the characteristics of a location other than latitude and longitude (e.g., inverse distance weighted method and ordinary kriging). By including chemical speciation of these particles, we are also the first, to our knowledge, to predict the spatial distribution of PM_10–2.5_ components. This is important because components can be used as indicators of different source types in the related health study.

This study has a few limitations. First, we used repeated 2-week samples over 1 year to assess long-term exposures to PM_10–2.5_. Although not an annual average, our sampling duration should be sufficiently long to mitigate transient perturbations such as transient meteorological fluctuations and reflect average conditions. Comparisons with available data from AQS monitors suggest that average PM_10–2.5_ mass during our 2-week sampling periods were highly correlated with, and within 15% of, the annual averages at the AQS stations. However, we cannot confirm our estimates for PM_10–2.5_ components because measured values are not available for comparison. Additionally, our models were derived based on data collected during a single year, and thus may not be accurate for other time periods if spatial patterns vary over time. Spatial stability has been recently demonstrated for traffic-related gases in Vancouver, Ontario, Canada, over 7 years (Wang et al. 2013) but is not guaranteed in other locations or for other pollutants. Other limitations pertain to our sampling locations. Although we attempted to capture different land uses, samples were collected at the homes of MESA participants, so our models may be more appropriate for predicting concentrations in residential areas than in industrial or commercial areas. In addition, our models may not have captured very small-scale spatial correlations, because only a few of the sampling locations were located within 100 m of one another. Finally, although each component targeted for this analysis was intended to be a common predictor of brake wear, tire wear, agriculture, or mineral dust across all cities, some caution is warranted in the strict interpretation of these indicator species because our tracers were not always unique to a single source (Sturtz et al. 2014). Research is ongoing to explore the spatial patterning of individual source contributions in further detail.

## Conclusions

In summary, we demonstrated that a spatially intensive monitoring campaign was useful in predicting fine-scale spatial variability of PM_10–2.5_ mass and chemical component concentrations within and across three U.S. cities. This research and the resulting prediction models represent a substantial improvement for epidemiology over studies of PM_10–2.5_ that have previously assigned pollutant concentrations from a central site to an entire city. That there are some differences in predictive performance by city and species, however, implies that caution should be taken in epidemiology studies when inferring the comparative health impacts of pollutants estimated by these types of models. Therefore, investigators should be mindful that any observed differences in apparent toxicity may also be at least partly attributable to differential accuracy in estimating concentrations.

## Supplemental Material

(330 KB) PDF
