# Erratum: “Characterizing Spatial Patterns of Airborne Coarse Particulate (PM_10–2.5_) Mass and Chemical Components in Three Cities: The Multi-Ethnic Study of Atherosclerosis”

**DOI:** 10.1289/ehp.122-A205

**Published:** 2014-08-01

In the original online version of “Characterizing Spatial Patterns of Airborne Coarse Particulate (PM_10–2.5_) Mass and Chemical Components in Three Cities: The Multi-Ethnic Study of Atherosclerosis” by Zhang et al. [Environ Health Perspect 122:823–830 (2014); doi:10.1289/ehp.1307287], Figures 1 and 2 were presented incorrectly as Figures 2 and 1, respectively. In addition, Tables 2 and 3 presented values for silicon in nanograms per cubic meter. The figures have been corrected, and the values for silicon in the two tables have been rescaled to micrograms per cubic meter.

The authors regret the errors.

