# Supplementary material for: Characterizing Spatial Patterns of Airborne Coarse Particulate (PM10–2.5) Mass and Chemical Components in Three Cities: The Multi-Ethnic Study of Atherosclerosis
Source: Environ Health Perspect. 2014 Mar 18;122(8):823–30. doi: 10.1289/ehp.1307287 (PMC4123025; doi:10.1289/ehp.1307287)
Supplement: (330 KB) PDF [file ehp.1307287.s001.pdf]

Environmental Health Perspectives doi:10.1289/ehp.1307287

Supplemental Material

Characterizing Spatial Patterns of Airborne Coarse Particulate ($PM_{10-2.5}$) Mass and Chemical Components in Three Cities: The Multi-Ethnic Study of Atherosclerosis

Kai Zhang, Timothy V. Larson, Amanda Gassett, Adam A. Szpiro, Martha Daviglus, Gregory L. Burke, Joel D. Kaufman, and Sara D. Adar

Table of Contents **Page**

Table S1. Detailed summary of statistics for $PM_{10-2.5}$ mass (μg/m^3 and chemical component (ng/m^3) concentrations at each sampling city by season 2

Table S2. Land use regression model estimates for the difference in mean $PM_{10-2.5}$ mass (μg/m^3) associated with a standard deviation difference in each predictor included in the final model for each city and for all three cities combined 4

Table S3. Land use regression model estimates for the difference in mean $PM_{10-2.5}$ copper (ng/m^3) associated with a standard deviation difference in each predictor included in the final model for each city and for all three cities combined 6

Table S4. Land use regression model estimates for the difference in mean $PM_{10-2.5}$ phosphorus (ng/m^3) associated with a standard deviation difference in each predictor included in the final model for each city and for all three cities combined 8

Table S5. Land use regression model estimates for the difference in mean $PM_{10-2.5}$ silicon (μg/m^3) associated with a standard deviation difference in each predictor included in the final model for each city and for all three cities combined 10

Table S6. Land use regression model estimates for the difference in mean $PM_{10-2.5}$ zinc (ng/m^3) associated with a standard deviation difference in each predictor included in the final model for each city and for all three cities combined 12

Table S7. Impacts of land use data sources on LUR model prediction performance for $PM_{10-2.5}$ mass concentrations 14

Table S1. Detailed summary of statistics for $PM_{10-2.5}$ mass (μg/m^3) and chemical component (ng/m^3) concentrations at each sampling city by season.

City and Season[a]	Sample size	Mean	SD[b]	Min	P25[c]	Median	P75[d]	Max
Chicago, IL - Total Mass								
Winter	33	5.54	1.98	1.95	4.43	5.32	6.27	10.69
Summer	31	5.94	2.09	2.84	4.51	5.40	7.86	11.73
Overall	64	5.73	2.03	1.95	4.48	5.36	6.87	11.73
St. Paul, MN - Total Mass								
Winter	25	3.34	2.22	-2.03	2.13	3.82	4.31	8.71
Summer	34	6.66	3.33	-5.53	5.09	6.37	7.73	17.12
Overall	59	5.25	3.33	-5.53	3.82	4.99	6.71	17.12
Winston-Salem, NC - Total Mass								
Winter	35	3.46	1.21	0.95	2.48	3.68	4.49	5.47
Summer	28	3.83	1.64	1.21	2.48	3.81	5.12	7.17
Overall	63	3.63	1.42	0.95	2.48	3.69	4.65	7.17
Chicago, IL - Copper								
Winter	32	7.83	3.32	1.62	5.42	7.20	10.08	13.50
Summer	31	7.10	4.37	-8.17	4.78	6.65	9.83	14.99
Overall	63	7.47	3.86	-8.17	5.29	7.15	10.00	14.99
St. Paul, MN - Copper								
Winter	25	4.01	1.23	2.42	3.38	3.64	4.49	7.89
Summer	34	2.77	1.69	0.59	1.84	2.43	2.97	9.10
Overall	59	3.29	1.63	0.59	2.17	2.97	3.81	9.10
Winston-Salem, NC - Copper								
Winter	35	2.57	1.23	0.83	1.71	2.31	3.36	5.99
Summer	28	2.57	1.46	-1.04	1.71	2.43	3.55	6.30
Overall	63	2.57	1.33	-1.04	1.71	2.41	3.39	6.30
Chicago, IL - Phosphorus								
Winter	32	13.64	6.00	2.11	10.54	12.72	15.93	32.98
Summer	31	17.87	3.87	11.26	15.33	16.95	20.16	26.57
Overall	63	15.72	5.46	2.11	12.20	15.48	18.75	32.98
St. Paul, MN - Phosphorus								
Winter	25	8.20	4.68	1.50	4.30	7.65	10.79	19.58
Summer	34	18.67	5.44	8.48	14.53	17.65	23.11	32.18
Overall	59	14.23	7.29	1.50	8.86	14.33	19.10	32.18
Winston-Salem, NC - Phosphorus								
Winter	35	12.83	3.70	5.07	10.43	13.40	15.29	22.41
Summer	28	25.90	5.71	11.04	22.76	25.97	29.04	38.08
Overall	63	18.64	8.04	5.07	12.78	16.44	25.18	38.08

City and Season[a]	Sample size	Mean	SD[b]	Min	P25[c]	Median	P75[d]	Max
Chicago, IL - Silicon								
Winter	32	428.24	105.37	217.53	357.74	430.10	501.98	633.57
Summer	31	306.84	157.92	92.32	205.19	263.37	408.88	793.36
Overall	63	368.51	146.16	92.32	259.38	360.55	463.32	793.36
St. Paul, MN - Silicon								
Winter	25	266.04	41.13	208.77	238.16	262.64	280.74	402.17
Summer	34	719.31	188.23	524.37	602.30	675.99	796.22	1339.88
Overall	59	527.25	268.13	208.77	265.82	558.41	704.48	1339.88
Winston-Salem, NC - Silicon								
Winter	35	410.63	85.93	285.46	354.55	382.39	461.41	748.54
Summer	28	345.90	109.79	172.76	287.69	330.06	382.41	661.99
Overall	63	381.86	101.74	172.76	323.22	370.12	433.24	748.54
Chicago, IL - Zinc								
Winter	32	23.74	18.36	2.38	11.87	19.36	24.99	69.20
Summer	31	25.87	22.85	-1.68	6.24	19.35	34.72	89.43
Overall	63	24.79	20.55	-1.68	11.68	19.35	32.73	89.43
St. Paul, MN - Zinc								
Winter	25	5.23	3.42	-1.58	3.15	5.28	7.42	14.38
Summer	34	5.55	7.03	-11.11	4.25	5.33	7.58	34.15
Overall	59	5.42	5.74	-11.11	3.62	5.28	7.58	34.15
Winston-Salem, NC - Zinc								
Winter	35	3.31	2.67	-5.55	1.95	3.67	4.81	7.27
Summer	28	2.76	1.95	-0.96	1.21	2.79	3.92	6.79
Overall	63	3.07	2.37	-5.55	1.84	3.46	4.74	7.27

[a]All sampling was conducted in 2009. Chicago: winter April 8–22, summer August 20–September 3; St. Paul: winter January 17–31, summer May 27–June 10; Winston-Salem: winter February 25–March 11, summer July 6–20. [b]Standard deviation. [c]25th percentile. [d]75th percentile.

Table S2. Land use regression model estimates for the difference in mean $PM_{10-2.5}$ mass ($\mu g/m^3$) associated with a standard deviation difference in each predictor included in the final model for each city and for all three cities combined.

**Covariates**	**Difference in predictor**	**Difference in $PM_{10-2.5}$ mass**
**Chicago, IL**		
Length of A2 road within 5 km buffer	1.29	-0.53 ± 0.22
Residential area within 15 km buffer	0.06	0.32 ± 0.20
Developed high intensity area within 0.75 km buffer	22.73	0.92 ± 0.23
Developed medium intensity area within 5 km buffer	10.33	0.71 ± 0.25
Residential area within 50 m buffer	0.46	-0.10 ± 0.21
Developed open space area within 3 km buffer	6.35	-0.28 ± 0.30
Summer	0.50	-0.25 ± 0.26
Summer × Residential area within 50 m buffer	0.42	-0.41 ± 0.26
Summer × Developed open space area within 3 km buffer	5.41	0.69 ± 0.25
**St. Paul, MN**		
Length of A1 road within 0.4 km buffer	0.06	1.10 ± 0.27
Transition area within 10 km buffer	0.002	-0.59 ± 0.28
Open water area within 0.5 km buffer	5.57	-0.80 ± 0.31
Developed medium intensity area within 3 km buffer	7.80	0.66 ± 0.32
Evergreen forest within 0.3 km buffer	1.60	0.20 ± 0.36
Non-forested wetland within 0.75 km buffer	1.08	-0.12 ± 0.41
Summer	0.50	1.28 ± 0.30
Summer × Evergreen forest within 0.3 km buffer	1.08	0.55 ± 0.35
Summer × Non-forested wetland within 0.75 km buffer	0.85	1.41 ± 0.41
**Winston-Salem, NC**		
Length of A3 road within 0.5 km buffer	0.13	0.41 ± 0.17
Developed low intensity area within 0.1 km buffer	29.72	0.38 ± 0.17
Evergreen forest within 0.3 km buffer	3.98	0.39 ± 0.18
Developed high intensity within 0.75 km buffer	4.22	0.31 ± 0.16
Shrub land area within 0.75 km buffer	0.74	-0.09 ± 0.23
Evergreen forest within 3 km buffer	2.34	0.18 ± 0.24
Summer	0.50	-0.25 ± 0.24
Summer × Shrub land area within 0.75 km buffer	0.63	-0.38 ± 0.25
Summer × Evergreen forest within 3 km buffer	2.24	0.88 ± 0.27

Covariates	Difference in predictor	Difference in $PM_{10-2.5}$ mass
**All cities**		
Distance to nearest large airport	0.60	-0.55 ± 0.20
Population density within 5 km buffer	1.12	0.91 ± 0.21
Log scale of distance to nearest railroad	1.20	-0.44 ± 0.15
Distance to nearest airport	0.48	-0.25 ± 0.21
Length of A1 road within 0.5 km buffer	0.09	0.27 ± 0.17
Log scale of distance to nearest A3 road	1.60	0.01 ± 0.17
Summer	0.50	0.66 ± 0.46
St. Paul	0.47	-0.56 ± 0.22
Summer × Distance nearest to airport	0.51	-1.41 ± 0.28
Summer × Log scale of distance to nearest A3 road	2.69	1.00 ± 0.46
St. Paul × Length of A1 road within 0.5 km buffer	0.06	0.62 ± 0.18

Table S3. Land use regression model estimates for the difference in mean $PM_{10-2.5}$ copper (ng/m^3) associated with a standard deviation difference in each predictor included in the final model for each city and for all three cities combined.

**Covariates**	**Difference in predictor**	**Difference in $PM_{10-2.5}$ copper**
**Chicago, IL**		
Log scale of distance to nearest A1 road	1.19	-1.14 ± 0.45
Length of A2 road within 0.4 km buffer	0.04	0.38 ± 0.40
CALINE long term average within 4.5 km buffer	4.34	0.38 ± 0.47
Developed high intensity within 0.3 km buffer	23.66	1.37 ± 0.41
Developed medium intensity within 5 km buffer	10.40	1.28 ± 0.34
Barren area within 1 km buffer	0.55	-0.55 ± 0.32
Summer	0.50	-1.04 ± 0.32
Summer × Length of A2 road within 0.4 km buffer	0.03	0.58 ± 0.40
**St. Paul, MN**		
Length of A1 road within 0.75 km buffer	0.16	0.46 ± 0.09
Length of A3 road within 5 km buffer	2.79	0.19 ± 0.09
Residential area within 50 m buffer	0.48	0.34 ± 0.08
Developed high intensity within 0.3 km buffer	14.43	0.72 ± 0.11
Developed open space area within 3 km buffer	6.29	0.14 ± 0.12
Summer	0.50	-0.41 ± 0.18
Summer × Nonforested wetland within 0.75 km buffer	0.85	0.81 ± 0.12
Summer × Developed open space area within 3 km buffer	8.11	-0.52 ± 0.20
**Winston-Salem, NC**		
Length of A3 road within 0.75 km buffer	0.21	0.45 ± 0.15
Log scale of distance to nearest A3 road	1.70	-0.17 ± 0.14
Commercial area within 0.5 km buffer	0.14	0.01 ± 0.16
Herbaceous rangeland within 0.5 km buffer	3.32	-0.31 ± 0.14
Shrub rangeland within 1 km buffer	0.69	-0.3 ± 0.13
Summer	0.50	-0.13 ± 0.15
Summer × Length of A2 road within 0.5 km buffer	0.02	0.45 ± 0.16
Summer × Commercial area within 0.5 km buffer	0.10	0.41 ± 0.17
**All cities**		
Population density within 10 km buffer	3.85	1.65 ± 0.22
Length of A1 road within 0.5 km buffer	0.09	0.47 ± 0.22
CALINE long term average within 3 km buffer	3.87	0.52 ± 0.22
Emissions of coarse particles within 30 km buffer	1.08	0.89 ± 0.24
Distance to nearest airport	0.48	-0.31 ± 0.2
Log scale of distance to nearest railroad	1.20	-0.22 ± 0.17
Summer	0.50	0.05 ± 0.25

Covariates	Difference in predictor	Difference in $PM_{10\text{-}2.5}$ copper
St. Paul	0.47	0.05 ± 0.8
Summer × Distance to nearest airport	0.51	-0.70 ± 0.27
St. Paul × Log scale of distance to nearest railroad	3.28	-1.32 ± 0.82

Table S4. Land use regression model estimates for the difference in mean $PM_{10-2.5}$ phosphorus (ng/m^3) associated with a standard deviation difference in each predictor included in the final model for each city and for all three cities combined.

**Covariates**	**Difference in predictor**	**Difference in $PM_{10-2.5}$ phosphorus**
**Chicago, IL**		
Distance to nearest large port	0.74	-4.6 ± 0.95
Industrial area within 0.75 km buffer	0.16	-0.82 ± 1.12
Transition area within 15 km buffer	0.01	0.28 ± 0.72
Developed medium intensity within 150 m buffer	24.01	-2.39 ± 0.60
Developed medium intensity within 5 km buffer	10.40	1.09 ± 0.66
Water area within 1 km buffer	10.16	-1.42 ± 0.61
Summer	0.50	-2.54 ± 1.26
Summer × Distance to nearest large port	0.83	3.54 ± 1.50
Summer × Industrial area within 0.75 km buffer	0.15	2.04 ± 1.16
Summer × Transition area within 15 km buffer	0.01	0.54 ± 0.92
**St. Paul, MN**		
Log scale of distance to nearest A1 road	1.06	0.45 ± 0.87
Mine area within 5 km buffer	0.002	-1.39 ± 0.60
Developed high intensity within 0.3 km buffer	14.43	1.82 ± 0.62
Forest land within 50 m buffer	16.08	0.8 ± 0.59
Pasture land within 0.75 km buffer	2.17	1.18 ± 0.83
Nonforested wetland within 0.75 km buffer	1.08	-0.1 ± 0.87
Summer	0.50	0.02 ± 3.50
Summer × Log scale of distance to nearest A1 road	3.34	4.82 ± 3.46
Summer × Pasture land within 0.75 km buffer	1.67	0.57 ± 0.84
Summer × Nonforested wetland within 0.75 km buffer	0.85	2.1 ± 0.86
**Winston-Salem, NC**		
Length of A1 road within 0.75 km buffer	0.11	1.74 ± 0.74
Length of A3 road within 0.75 km buffer	0.21	1.71 ± 0.62
Log scale of distance to nearest truck route	0.96	0.85 ± 0.81
Commercial area within 105 m buffer	0.20	0.37 ± 0.65
Mixed forest land within 0.75 km buffer	0.53	0.89 ± 0.63
Summer	0.50	-4.53 ± 3.68
Summer × Commercial area within 105 m buffer	0.14	0.41 ± 0.69
Summer × Mixed forest land within 0.75 km buffer	0.35	1.17 ± 0.66
Summer × Log scale of distance to nearest truck route	3.63	11.04 ± 3.72

**Covariates**	**Difference in predictor**	**Difference in $PM_{10-2.5}$ phosphorus**
**All cities**		
Length of A3 road within 1.5 km buffer	0.70	1.43 ± 0.43
Length of A2 road within 5.0 km buffer	1.07	-1.17 ± 0.4
75th percentile NDVI within 10 km buffer	37.89	-0.22 ± 0.56
Summer	0.50	-4.52 ± 1.53
Log scale of distance to nearest rail road	1.20	-0.24 ± 0.48
St. Paul	0.47	4.45 ± 2.28
Summer × 75th percentile NDVI within 10 km buffer	84.18	9.51 ± 1.56
St. Paul × Log scale of distance to nearest rail road	3.28	-6.39 ± 2.28

Table S5. Land use regression model estimates for the difference in mean $PM_{10\text{-}2.5}$ silicon ($\mu g/m^3$) associated with a standard deviation difference in each predictor included in the final model for each city and for all three cities combined.

**Covariates**	**Difference in predictor**	**Difference in $PM_{10\text{-}2.5}$ silicon**
**Chicago, IL**		
Residential area within 50 m buffer	0.30	-0.05 ± 0.01
Residential area within 15km buffer	0.06	0.06 ± 0.02
Other urban are within 15 km buffer	0.01	-0.02 ± 0.02
Developed high intensity within 0.3 km buffer	23.66	0.04 ± 0.01
Developed open space area within 0.4 km buffer	8.51	-0.03 ± 0.02
Water area within 0.75 km buffer	7.65	-0.01 ± 0.02
Summer	0.50	0.05 ± 0.05
Summer × Other urban are within 15 km buffer	0.02	-0.13 ± 0.05
Summer × Water area within 0.75 km buffer	5.34	-0.03 ± 0.02
**St. Paul, MN**		
Developed high intensity within 0.4 km buffer	13.60	0.02 ± 0.01
Other urban are within 0.5 km buffer	0.09	0.002 ± 0.01
Evergreen forest land within 3 km buffer	0.50	-0.02 ± 0.01
Shrub rangeland within 0.5 km buffer	0.88	0.02 ± 0.01
Nonforested wetland within 0.75 km buffer	1.08	-0.004 ± 0.01
Summer	0.50	0.19 ± 0.01
Summer × Developed high intensity within 0.4 km buffer	11.46	0.04 ± 0.01
Summer × Other urban are within 0.5 km buffer	0.06	-0.04 ± 0.01
Summer × Shrub rangeland within 0.5 km buffer	0.71	0.02 ± 0.01
Summer × Nonforested wetland within 0.75 km buffer	0.85	0.10 ± 0.01
**Winston-Salem, NC**		
Length of A3 road within 0.5 km buffer	0.13	0.03 ± 0.01
Industrial area within 1.5 km buffer	0.04	0.001 ± 0.01
Open water area within 0.75 km buffer	0.53	-0.02 ± 0.01
Evergreen forest within 0.3 km buffer	3.98	0.03 ± 0.01
Pasture land within 0.3 km buffer	9.24	-0.02 ± 0.01
Shrubland area within 0.75 km buffer	0.74	-0.02 ± 0.01
Summer	0.50	-0.05 ± 0.01
Summer × Industrial area within 1.5 km buffer	0.03	0.04 ± 0.01
Summer × Pasture land within 0.3 km buffer	6.46	0.05 ± 0.01
Summer × Shrubland area within 0.75 km buffer	0.63	-0.01 ± 0.02

**Covariates**	**Difference in predictor**	**Difference in $PM_{10-2.5}$ silicon**
**All cities**		
Length of A3 road within 50 m buffer	0.00	-0.02 ± 0.01
Log scale of distance to nearest rail road	1.20	-0.04 ± 0.01
Distance to nearest airport	0.48	0.02 ± 0.02
Length of A1 road within 3 km buffer	0.98	0.02 ± 0.01
CALINE long term average within 9 km buffer	4.02	-0.02 ± 0.02
Emissions of coarse particles within 3 km buffer	0.01	-0.05 ± 0.02
Summer	0.50	0.11 ± 0.03
St. Paul	0.47	0.02 ± 0.02
Summer × Distance to nearest airport	0.51	-0.14 ± 0.02
Summer × CALINE long term average within 9 km buffer	3.73	0.03 ± 0.02
Summer × Emissions of coarse particles within 3 km buffer	0.01	0.07 ± 0.02
St. Paul × Length of A1 road within 3 km buffer	0.88	0.04 ± 0.02

Table S6. Land use regression model estimates for the difference in mean $PM_{10-2.5}$ zinc (ng/m^3) associated with a standard deviation difference in each predictor included in the final model for each city and for all three cities combined.

Covariates	Difference in predictor	Difference in $PM_{10-2.5}$ zinc
**Chicago, IL**		
Length of A1 road within 0.5 km buffer	1.16	11.2 ± 1.92
Length of A2 road within 3 km buffer	0.62	-2.89 ± 1.60
Industrial area within 1km buffer	0.16	10.41 ± 1.59
Developed open space area within 3 km buffer	6.39	-3.21 ± 1.76
Developed high intensity within 0.3 km buffer	23.66	-0.13 ± 2.39
Forest land within 0.4 km buffer	4.04	2.41 ± 1.41
Summer	0.50	-2.05 ± 1.87
Summer × Developed high intensity within 0.3 km buffer	21.27	-4.09 ± 2.33
**St. Paul, MN**		
Distance to nearest A2 road	0.12	-1.46 ± 0.55
Transport are within 0.5 km buffer	0.06	0.68 ± 0.61
Commercial area within 0.75 km buffer	0.19	1.02 ± 0.64
Transition area within 1.5 km buffer	0.03	0.03 ± 0.74
Shrub rangeland within 3 km buffer	0.68	1.41 ± 0.55
Evergreen forest within 0.4 km buffer	1.16	0.14 ± 0.74
Summer	0.50	0.12 ± 0.58
Summer × Transition area within 1.5 km buffer	0.02	3.42 ± 0.71
Summer × Evergreen forest within 0.4 km buffer	0.84	-1.53 ± 0.72
**Winston-Salem, NC**		
Transport are within 1 km buffer	0.05	0.67 ± 0.26
Residential area within 50 m buffer	0.47	0.34 ± 0.31
Other urban are within 0.75 km buffer	0.05	-0.84 ± 0.24
Mixed forest land within 5 km buffer	0.45	-0.57 ± 0.25
Shrub rangeland within 1 km buffer	0.69	-0.53 ± 0.27
Open water area within 5 km buffer	0.72	-0.04 ± 0.38
Summer	0.50	0.38 ± 0.41
Summer × Residential area within 50 m buffer	0.42	-1.21 ± 0.41
Summer × Open water area within 5 km buffer	0.63	0.82 ± 0.41
**All cities**		
Population density within 10 km buffer	3.85	13.50 ± 1.33
Length of A2 road within 5 km buffer	1.07	-3.16 ± 0.78
Log scale of distance to nearest truck route	1.09	-2.15 ± 0.75
Distance to nearest airport	0.48	-2.36 ± 1.16
Length of A3 road within 0.5 km buffer	0.12	-0.74 ± 1.01

**Covariates**	**Difference in predictor**	**Difference in $PM_{10-2.5}$ zinc**
Length of A3 road within 5 km buffer	5.80	-0.25 ± 1.42
Summer	0.50	1.91 ± 1.62
St. Paul	0.47	1.62 ± 2.84
Summer × Distance to nearest airport	0.51	-2.07 ± 1.41
Summer × Length of A3 road within 0.5 km buffer	0.11	-1.90 ± 1.22
St. Paul × Length of A3 road within 5 km buffer	6.34	-4.20 ± 2.81

Table S7. Impacts of land use data sources on LUR model prediction performance for $PM_{10-2.5}$ mass concentrations.

City	CV Measure	2000 + Supplemental 1970s/1980s	2000	1970s/1980s
Chicago, IL	R^2	0.68	0.68	0.64
Chicago, IL	RMSE	1.16	1.16	1.22
St. Paul, MN	R^2	0.51	0.49	0.39
St. Paul, MN	RMSE	2.33	2.37	2.60
Winston-Salem, NC	R^2	0.41	0.41	0.36
Winston-Salem, NC	RMSE	1.09	1.09	1.14
